# Effect of Putrescine Inoculation In Ovo on Hatchability, Hepatic and Muscular Glycogen Reserve, Intestinal Morphology, and Performance of Broilers

**DOI:** 10.3390/ani15091259

**Published:** 2025-04-29

**Authors:** Katiucia Cristine Sonálio, Leopoldo Malcorra de Almeida, Lucas Schmidt Bassi, Leandro Nagae Kuritza, Isabela de Camargo Dias, Chayane da Rocha, Alex Maiorka

**Affiliations:** 1 Department of Veterinary Science, Federal University of Paraná, Curitiba 80035-050, PR, Brazil; almeidamleopoldo@gmail.com (L.M.d.A.); leandronkmv@gmail.com (L.N.K.); isabellacdias24@gmail.com (I.d.C.D.); amaiorka@ufpr.br (A.M.); 2 Department of Animal Science, Federal University of Paraná, Curitiba 80035-050, PR, Brazilchayane.ufpr@gmail.com (C.d.R.)

**Keywords:** embryonic development, embryo nutrition, 1,4-diaminobutane, intestinal development

## Abstract

The objective of this study was to evaluate the inoculation of nutrient solutions with increasing levels of putrescine on the hatchability, physiology, and performance of broilers during the initial phase. The study is composed of four treatments with increasing doses of putrescine (0.015; 0.030; 0.060, and 0.090%) and a control group. At hatch, hatchability rate; ratio between egg weight and chick weight; ratio between yolk sac, liver, breast and intestine weight, and chick weight; glycogen concentration in the liver and breast; and morphometric characteristics of the jejunum and ileum were evaluated. After hatch, 400 birds were housed in metabolic cages according to the treatments received, and feed intake, body weight gain, and feed conversion ratio were assessed at specific time points. Hatchability, chick performance at hatch, and organ weight were not affected by the inoculation of increasing levels of putrescine. Intestinal villi at hatch were higher in groups supplemented with putrescine (*p* < 0.001). The amounts of hepatic glycogen per gram of tissue at hatch were higher in groups with the lowest levels of putrescine and decreased with increasing doses (*p* = 0.017). Growth performance from 1 to 21 days was not influenced by the inoculation of putrescine.

## Simple Summary

This study explores how putrescine, a compound involved in cell growth, affects the development and early growth of broiler chickens. Researchers added different amounts of putrescine to the nutrient solutions given to chicken embryos and observed the effects on various aspects like hatchability, body weight, organ size, and gut development. They also looked at the levels of glycogen, an energy source, in the liver and breast muscles. The results show that while putrescine did not significantly change the number of chicks hatched or their early growth, it did result in taller gut-lining structures, which are important for nutrient absorption. Additionally, lower doses of putrescine were associated with higher energy reserves in the liver. The findings suggest that putrescine can enhance intestinal development without affecting overall growth or hatch success, potentially benefiting poultry farming by improving nutrient absorption and health in young birds. This could lead to more efficient and healthier poultry production, offering benefits to farmers and consumers alike by supporting the development of more robust and resilient chickens.

## 1. Introduction

During incubation, embryo development is complete if the egg contains all necessary nutrients, though their quantity and quality may impose limitations [1,2]. Soon after hatching, birds must be able to digest and absorb the nutrients provided by the diet to achieve maximum productivity and growth [3]. However, in the first days of life, the chicks’ gastrointestinal tract (GIT) is not yet physiologically developed and has limited digestive functions, which can decrease the use of nutrients for their growth [4]. To overcome this, in ovo feeding (IOF) of substances can provide specific compounds to anticipate the development of the GIT; increase glycogen reserves; reduce mortality; and increase the growth rate, feed efficiency, and overall health of birds [5,6,7].

Putrescine is a compound with the potential to be fed in ovo to aid in the development embryos of birds. It is a polyamine with low molecular weight synthesized from ornithine through ornithine decarboxylase (ODC), which acts as the rate-limiting step in the biosynthesis of putrescine [8,9,10]. Putrescine has a simple chemical structure (NH_2_(CH_2_)_4_NH_2_) and is soluble in water [11,12]; thus, it is easily metabolized by the embryo and suitable for administration in ovo [13]. Key roles of putrescine in the organism include: regulation of the synthesis of nucleic acids; modulation of DNA structure and mRNA translation; and essential functions in cell proliferation, growth, and differentiation [8,9,14,15,16]. Considering that cell proliferation and differentiation are important for maintaining mucosal morphology and its function [9], it is important to know that putrescine supplementation can promote the early development of the GIT, increasing bird growth rates [10,17].

Putrescine also plays an essential role in the small intestine by forming succinate via gamma-aminobutyric acid (GABA), serving as a readily available source of instant energy metabolization [18]. In the previous study, the authors assessed an intragastrical administration of putrescine in fasted rats and observed that even though putrescine concentrations in the small intestine tissues decreased during fasting, the administration increased the uptake from circulation in the jejunum, and more than 80% of it was immediately converted to succinate. Thus, putrescine can become available through the systemic circulation, providing rapid metabolizable energy to the intestine [18].

Putrescine was tested as a nutrient in diets for broiler chickens in a study conducted by Smith et al. [17] and Qi et al. [10], where the dietary inclusion of putrescine resulted in a higher growth rate, decreased the ratio of feed, higher percentage of intestine weight in relation to the animal’s body weight and heavier birds in the growth phase. In a study, Goes et al. [13] evaluated putrescine for the first time as a component of in ovo nutritional solutions and observed a trophic effect that favored intestinal weight immediately post- hatch. However, the authors also observed an increase in the number of goblet cells at 2 days post-hatch, which may have negatively affected the rate of feed conversion during the first week.

Based on the above considerations, this study evaluates the effects of nutrient solutions containing increasing levels of putrescine inoculated in ovo on incubation performance, intestinal morphometry, hepatic and muscle glycogen concentration at birth and in the post-hatch period, in addition to evaluating the animal’s performance during the initial phase. Because there is no record of the toxic limit of putrescine for bird embryos in the literature, based on the study by Goes et al. [13], tested doses were below 0.1% of putrescine in ovo, to better understand the effects of this substance on embryos of broilers.

## 2. Materials and Methods

This study was approved by the Ethics Committee on Animal Use (CEUA SCA) of the Federal University of Paraná, under protocol number 017/2019, dated April 2019. The present study had a total duration of 42 days, with the initial 21 days necessary for the incubation period and the 21 days after birth necessary to assess the performance of the birds.

### 2.1. Egg Selection and Incubation

A total of 640 eggs from a 34-week-old breed of (Ross^®^ 308, Aviagen, Huntsville, AL, USA) broiler breeders, from a commercial breeder, were incubated. The eggs were weighed individually, and those with an average weight of 64.42 ± 0.82 g, free of cracks, deformities or dirt, were selected. Before entering the hatchery, the eggs were disinfected with a 2:1 solution of formaldehyde (40%) and potassium permanganate (KMnO_4_) (16 mL of formaldehyde to 8 g of potassium permanganate m^2^ of air space) and remained in the storage room for three days under a constant temperature of 18 °C.

On the day of incubation, the eggs were preheated for 6 h at a temperature of 26 °C and humidity of 65%, distributed in the incubator (Avicomave^®^, Iracemápolis, Brazil), and maintained at a temperature of 37.5 °C and 55% humidity until the time of inoculation, at 17 days. To ensure that all eggs were inoculated at the same moment of embryonic development, at the beginning of the incubation process the trays were inserted into the incubator with a 60 min interval between them.

The eggs were distributed in a completely randomized design with 5 treatments consisting of 4 nutrient solutions containing increasing doses of putrescine (PUT 0.015, PUT 0.030, PUT 0.060, and PUT 0.090%) and a control group inoculated with sodium chloride solution (NaCl; 0.9%). Each treatment consisted of 128 eggs divided into 8 replicates of 16 eggs each. On the 10th day of incubation, the eggs were submitted to ovoscopy for the disposal of infertile eggs and early embryonic mortality.

### 2.2. Preparation and Injection of IOF Solutions

The putrescine-based nutrient solutions were prepared in a laboratory, with sterile equipment and utensils, under a laminar flow hood to avoid microbiological contamination. To prepare the solutions, putrescine (>99.0% purity; Sigma^®^, São Paulo, Brazil) was diluted in sterile saline (0.90%). The pH and osmolarity of the solutions were monitored with a pH meter (Gehaka-PG1800, São Paulo, Brazil) and an osmometer (Knauer-K7400S, Berlin, Germany), respectively, as pH below 4 and osmolarity above 800 Osm can interfere and hinder the embryonic development [19]. The pH values of the solutions with increasing concentrations of putrescine (0.015, 0.030, 0.060and 0.090%) were 8.43, 9.64, 9.93, and 10.26 respectively. Osmolarity results were 285 mmol/kg in the 0.015 solution, 296 mmol/kg in the 0.030 solution, and 297 mmol/kg in the 0.060 and 0.090% putrescine solutions

The in ovo injection procedure for the solutions was based on Uni and Ferket [19] with some modifications. Briefly, inoculation of the solutions occurred on the 17th day of incubation (408 h), in a cleaned and heated room with a constant temperature of 36 °C. The solutions were kept in a water bath at 37.5 °C to prevent thermal variations from affecting the embryo at the time of inoculation. The eggs were removed from the incubator and taken to the inoculation room, where ovoscopy was performed to observe the position of the embryo, amniotic fluid, and larger caliber vessels. After being disinfected with 70% alcohol, the eggshell was punctured in the region of the air chamber with the aid of a stainless-steel manual perforator (Kitchen Craft^®^ Birmingham, United Kingdon). Then, 0.5 mL of the solution was injected directly into the amniotic fluid of each embryo according to its corresponding treatment using 3 mm needles and 1 mL syringes. Immediately after inoculation, the egg orifice was sealed with beeswax, and the eggs were transferred to the hatchers (Avicomave^®^, Iracemápolis, Brazil) and kept at 36.8 °C with 68% humidity until hatching.

### 2.3. Incubation Rate

The hatchers were opened after 504 h of incubation (21 days) and all chicks were removed. The chicks were then individually weighed and sexed by observing the warping of the birds’ wings according to its lineage manual [20]. The ratio between egg weight and chick weight was determined by the average weight of the animals at birth and the egg weight before incubation: (average chick weight (g) × 100)/average egg weight (g).

Shortly after hatch, 6 birds from each treatment were randomly selected and euthanized by cervical dislocation. Then, yolk, liver, intestine, and breast were weighed with a precision scale (Bioprecisa^®^-FA2104N, Curitiba, Brazil) to calculate their relative weight in relation to the body weight of the chick (g of tissue/g of body weight) × 100.

At the end of the incubation period, an embryo diagnosis was carried out, where the eggs that did not hatch were opened individually and classified according to the stage of embryonic development at the time of death [21,22]. At the same time, edema, bleeding, and/or malformations were described. The hatchability rate was calculated by the number of hatched chicks in relation to the number of fertile eggs and expressed as a percentage.

### 2.4. Analysis of Hepatic and Muscle Glycogen

The assessment of hepatic and muscular glycogen was performed according to the colorimetric method described by Hassid and Abraham [23] and Dal Pont et al. [24] and expressed in mg/g of wet tissue. For this, the liver and the pectoral muscle of 6 chicks per treatment were collected immediately after hatching, weighed, and immediately frozen in liquid nitrogen, and stored in a freezer at −20 °C until the time of analysis.

### 2.5. Analysis of Intestinal Morphology

The analysis of morphometric variables of the intestinal mucosa was performed at birth and on the 4th day post-hatching. On each period, 2 cm fragments of the jejunum (2 cm above the yolk diverticulum) and the ileum (2 cm below the yolk diverticulum) were collected from 6 randomly selected birds per treatment. Samples of the intestinal segments were sectioned longitudinally, spread on firm paper, stapled by the ends, washed carefully with 0.90% saline solution, and fixed in 10% buffered formalin. The histological sections were prepared, fixed on slides, and stained with hematoxylin-eosin and alcian blue. The reading of the intestinal portion slides was performed through an optical microscope (LEICA^®^-DM500, Wetzlar, Germany) coupled with a camera (TOUP-CAM^®^ Hangzhou, China). with image capture by the program (TOUPVIEW^®^, Hangzhou, China). Ten villi and crypts per bird were evaluated to determine villus height (VH), crypt depth (CD), and goblet cell count. The height of the villi was measured from the basal region, which coincides with the upper portion of the crypts up to their summit, and the crypts, from the base to the transition region crypt: villus. Goblet cells were counted over 200 μm, in the intermediate portion of the villus [25].

### 2.6. Growth Performance

For the performance evaluation, 400 sexed chicks were housed until 21 days of age, assigned to five inoculation treatments, and divided into 8 replicates of 10 animals each, blocked by gender. The chicks were housed in metabolic cages (0.98 m long × 0.45 m wide × 0.50 m high), equipped with gutter feeders and drinkers. The environmental control of the experimental room followed the specifications described in the Ross broiler management [26]. The feed provided was identical for all treatments based on corn and soybean meal, according to the nutritional requirements of the Brazilian Tables for Poultry and Swine [27] for the initial breeding phase (Table 1), and offered in mashed form.

The performance variables were obtained weekly (1, 7, 14, and 21 days) by weighting the groups of birds, feed offer and feed leftovers, to calculate the average feed intake (FI), average body weight gain (BWG), and feed conversion ratio (FCR) in the accumulated period, corrected for the weight of dead birds.

### 2.7. Statistical Analysis

The incubation data were analyzed according to a completely randomized design. Performance data were analyzed according to a randomized block design, as birds were blocked by gender and each cage of 10 birds was considered an experimental unit. All data collected were subjected to analysis of homogeneity of variances and residue normality. Data with normal distribution were then subjected to ANOVA, and in the effect of increasing putrescine doses, data were analyzed by linear and polynomial regression. Values with *p* ≤ 0.05 were considered statistically significant and values with *p* < 0.05 to *p* ≤ 0.1 were considered marginally statistically significant.

## 3. Results

The hatchability rate was not affected by putrescine in ovo inoculation (*p* = 0.634) as described in Table 2. The embryo mortality after solution injections was 25.20% in the control treatment; 19.35% in PUT 0.015; 27.27% in PUT 0.030; 24.19% in PUT 0.060; and 28.45% at PUT 0.090%.

The ratio of egg weight and chick weight were not affected by the different treatments (*p* = 0.210). Results were 69.32%, 70.53%, 67.80%, 71.34%, and 69.68% for the control treatment (0.9% saline) PUT 0.015, PUT 0.030, PUT 0.060, and PUT 0.090%, respectively. Chick weight at birth did not differ between treatments (*p* = 0.353). The variables of yolk weight (*p* = 0.289), liver (*p* = 0.686), breast (*p* = 0.879), and intestine (*p* = 0.489) in relation to the chick’s body weight at hatch did not show significant difference between treatments. At 4 days after hatch, the body weight of the bird (*p* = 0.723) did not differ statistically. However, the percentage of intestine weight in relation to the body weight of the chick (*p* = 0.054) was marginally statistically significant, with values numerically higher in treatments with PUT 0.015 and PUT 0.060% (Table 3).

A linear effect (*p*-linear = 0.017) on the concentration of hepatic glycogen was detected, as it decreased with increasing putrescine doses (Table 4). A quadratic effect (P-quadratic = 0.022) was observed for pectoral muscle glycogen in response to the increasing levels of putrescine.

In addition, putrescine doses influenced the intestinal morphometry of the jejunum at hatch, as VH showed an increasing linear effect (*p* < 0.001) with increasing putrescine doses, the highest VH values were observed (332.98 µm and 318.42 µm) in PUT 0.060 and PUT 0.090% treatments, respectively. The CD was greater in the treatment with PUT 0.090 and PUT 0.060% (72.20 µm and 71.58 µm, respectively), as a quadratic effect (*p* < 0.001) was observed with increasing levels of putrescine. The number of goblet cells linearly increased (*p* < 0.001) with higher putrescine levels, demonstrating that the highest numbers were obtained in the PUT 0.090 (17.91 cells in 200 µm) and PUT 0.060% (17.72 cells in 200 µm) treatments, respectively.

In the period of 4 days post-hatch, VH showed a quadratic effect (*p* < 0.001), where the highest value (484.50 µm) was observed in the PUT 0.030% treatment and the lowest value (418.87 µm) in the PUT 0.090% treatment. Crypt depth decreased linearly (*p* < 0.001) according to the increase in putrescine levels, with the highest values observed in the control treatments and 0.015% (126.54 µm and 125.94 µm, respectively), and the lowest value was found in the PUT treatment 0.060% (105.65 µm). The number of goblet cells linearly increased with higher putrescine doses (*p* < 0.001); the lowest number of cells was observed in the negative control treatment (22.60 cells in 200 µm) and the highest number in PUT treatment 0.090% (27.66 cells in 200 µm). All data on the morphological evaluation of the jejunum are shown in Table 5.

As for the morphological evaluation of the ileum (Table 6), there was a quadratic effect of putrescine doses on VH (*p* < 0.001), CD (*p* = 0.034) and number of goblet cells (*p* = 0.023) at hatch. At 4 days post-hatch, VH linearly increased (*p* ≤ 0.001) and CD linearly decreased (*p* = 0.008) with increasing putrescine doses. The number of goblet cells had a quadratic response (*p* < 0.001) to the increase in putrescine levels, with the lowest value observed in the control treatment (25.88 cells in 200 µm) and the highest values (32.45 and 30.64 cells in 200 µm) observed in the treatments PUT 0.030 and PUT 0.090%, respectively.

In the growth performance evaluation, average FI, average BWG, and FCR in the periods from 1 to 7, 1 to 14, and 1 to 21 days showed significant differences between treatments (Table 7).

## 4. Discussion

The hatchability rate was not affected by the inoculation of putrescine solutions in ovo. In this study, doses of putrescine below 0.1% were used, as recommended by Goes et al. [13], who observed a lower rate of hatchability in treatments with the inoculation of putrescine in ovo—a fact that may be related to the toxicity of putrescine to embryos in doses higher than 0.1% (0.15 and 0.2%).

The osmolarity of putrescine solutions remained below 800 Osm which, according to [19], are suitable for solutions to be inoculated in ovo without causing physiological damage to the embryos and consequently impairing hatchability. The pH of the solutions showed higher alkalinity when putrescine doses were increased; In the egg, pH fluctuates during embryonic development according to the stage of development and to the substrates involved in the embryo metabolism. An interaction occurs between blood, amnion and allantoic fluid to regulate the pH of both blood and amnion, which remain constant, while the allantoic pH decreases [28,29]. The pH of amnion seems to be buffered by the amount of HCO_3_^−^ in the allantois, which acts both as a source of respiratory HCO_3_^−^ and as a site of acidification processes; From the 14th day on, the level of amniotic HCO_3_^−^ decreases [29,30]. Therefore, this ability to maintain pH balance during embryonic development may be a possible reason why the embryo restores basic-acid balance after inoculation of alkaline solutions, without impairing the hatchability rate.

The results observed for the ratio between egg weight and chick weight at hatch corroborate the results found by Goes et al. [13] as the authors reported that different concentrations of putrescine (0.05, 0.1, 0.15, and 0.2%) did not differ from the control treatment for egg weight/chick weight ratio at hatch.

The percentages of breast and liver weight in relation to the chick’s body weight at hatch corroborate the results found by Goes et al. [13], who also reported no difference between the different doses of putrescine used. However, the results of the percentage of intestines in relation to the chick’s weight differed from Goes et al. [13] who observed a higher percentage of intestine weight in relation to body weight in birds inoculated with 0.15% putrescine, a higher dose than those evaluated in this study.

In a study with putrescine supplementation in the initial diet for turkeys, Smith et al. [17] assessed the effect of 0.2, 0.4, and 0.6% putrescine levels and observed that birds fed with 0.2% of putrescine had higher growth rate and intestinal weight percentage in relation to the animals’ body weight, concluding that early development of the GIT results in higher rates of body growth, especially in the grower phase. The greater weight of the intestine in these studies can be attributed to the influence of putrescine on cell proliferation, growth, and differentiation in the intestinal mucosa [9,10,31]. However, when putrescine was inoculated in ovo, the effect of intestinal weight gain was not observed at 24 h [13] or 96 h post-hatch (current study). A hypothesis for this lack of effect may be due to the intense transformation that occurs in the intestine after hatching and during the first days of the chick’s life, with major changes in the enterocyte profile which rapidly adapts to an exogenous diet [31,32,33].

Supplementation in ovo with putrescine resulted in greater VH and CD in the jejunum and ileum of chicks at hatch; again, the effects of polyamine supplementation are known to promote cell proliferation, including intestinal cells [9,17,34]. This occurs through the involvement of polyamines in signal transduction and in almost all stages of DNA, RNA, and protein synthesis. Because cell renewal in the intestinal epithelium is greater than in most other parts of the body, polyamines can be considered vital for a proper structure and function of the GIT [8,16].

Goblet cells act in the maintenance and development of the intestinal epithelium, as they secrete glycoprotein mucins whose primary function is to protect the intestinal epithelium from the action of digestive enzymes and abrasive effects of the digesta during in ovo development and after hatching [35]. In this study, the increase in goblet cells observed in birds supplemented with putrescine solutions is similar to that reported by Goes et al. [13], where the concentrations of putrescine used for in ovo feeding showed a higher number of goblet cells, suggestive of an intestinal tissue response to the possible toxic effect of putrescine for the embryo.

In the study conducted by Goes et al. [13], the authors observed a higher percentage of breast muscle in birds supplemented with putrescine, a fact that could be related to putrescine’s ability to readily make energy available to the intestine, among other organs, saving energy from other sources used during the final stage of embryonic development, such as glycogenic amino acids in the breast muscle [36,37], thus preventing tissue loss. Bardócz et al. [18] observed that polyamines can be catalyzed in the form of an acid known as gamma-aminobutyric acid, which is subsequently converted to succinate and incorporated into the Krebs cycle. Because of this, polyamines may serve as a source of instant energy metabolization, supporting the metabolic needs of intestinal tissue and possibly increasing glycogen levels in the liver and muscles. In the current study, it was observed that the amount of hepatic glycogen was higher in treatments with lower levels of putrescine supplementation (PUT 0.015 and 0.030%), decreasing linearly with the addition of putrescine in the solutions. Glycogen in the breast muscle was similarly affected, as the PUT treatments 0.015, 0.030, and 0.060% presented the highest amount of glycogen per gram of tissue. However, the higher concentration of putrescine did not have the same effect of saving energy in the form of hepatic glycogen or breast muscle, which may be associated with a negative/toxic effect of higher concentrations of putrescine (PUT 0.090%), which goes in accordance with the results reported by Goes et al. [13] who reported toxic effects at doses greater than 0.1%.

Furthermore, the results of this study showed no differences in growth performance between treatments during the evaluated periods. In contrast, Goes et al. [13] observed higher feed conversion rates in broilers fed with higher-concentration in ovo putrescine solutions. This may be related to a greater energy expenditure to meet the energy demands for the following: intestinal maturation; villous growth; mucin production; and maintenance of intestinal tissue. On the other hand, Smith et al. [17] argue that putrescine supplementation can promote the growth of turkeys in the initial period and that this early development of the GIT results in an increase in the growth rates of birds during the rearing phase.

The evaluated doses of in ovo putrescine did not affect bird performance, likely due to physiological changes occurring in the gastrointestinal tract (GIT) during the post-hatch period. During this time, intense proliferation of new enterocytes takes place at the Lieberkühn crypts, migrating to the tips of the villi [33]. This can alter the effect of putrescine on the subsequent performance because these enterocytes develop a selective tendency towards the digestion of nutrients coming from exogenous diets [3,33,38].

Lastly, it is important to mention that the post-hatch period is highly complex and marked by significant changes in GIT morphology. Additionally, these changes may be influenced not only by the concentration of putrescine but also by the pH of the different solutions. Thus, a deeper understanding of the interaction between putrescine and the transition from embryo egg-based to diet-based nutrition is needed, particularly regarding its effects on GIT development, FI, and overall broiler performance in the initial phase.

## 5. Conclusions

Incubation yield and performance were not affected by the use of putrescine in ovo at the levels of 0.015, 0.030, 0.060, and 0.090%. The inoculation of putrescine in ovo promoted higher intestinal villi of chicks at hatch.

## Figures and Tables

**Table 1:** Ingredients and nutritional levels of the experimental diet.

Ingredients (%)	Initial Diet
Corn	57.063
Soybean meal (46% crude protein)	38.500
Soybean oil	1.000
Dicalcium phosphate	1.000
Limestone	1.170
Salt (NaCl)	0.510
DL-methionine	0.264
L-lysine	0.111
L-threonine	0.032
Choline chloride	0.100
Vitamin premix ^†^	0.130
Mineral premix ^‡^	0.050
Phytase ^§^	0.015
Carbohydrase ^¶^	0.005
Calculated nutritional composition	Amounts
Metabolizable energy (kcal/kg)	2947
Crude protein (%)	21.994
Ether extract (%)	3.758
Crude fiber (%)	2.507
Calcium (%)	0.893
Available phosphorus (%)	0.448
Sodium (%)	0.218
Chlorine (%)	0.379
Digestible lysine (%)	0.568
Digestible methionine (%)	0.888
Digestible methionine + cystine (%)	0.570
Digestible cystine (%)	0.236
Digestible arginine (%)	1.199
Digestible tryptophan (%)	0.779
Digestible threonine (%)	0.915
Digestible valine (%)	0.880
Digestible isoleucine (%)	0.740

^†^ Supplied per kilogram of diet: vitamin A, 14.43 UI; vitamin D3, 234.06 UI; vitamin E, 19.48 ppm; vitamin K3, 10.16 ppm; vitamin B1, 31.20 ppm; vitamin B2, 15.56 ppm; pantothenic acid 0.78 ppm; vitamin B6, 0.058 ppm; vitamin B12, 7.78 ppm; nicotinic acid, 78.03 ppm; folic acid, 194.93 ppm; biotin, 1596.39 ppm. ^‡^ Supplied per kilogram of diet: selenium, 0.225 ppm; copper sulfate, 7.50 ppm; iron sulfate, 30.00 ppm; iodine, 0.513 ppm; manganese oxide, 45.00 ppm; zinc oxide, 45.00 ppm. ^§^ Ronozyme Hiphos (CT) 20.000 FYT/g of product. ^¶^ Ronozyme HiStarch (CT) 80 KNU/g of product.

**Table 2:** Effect of putrescine in ovo on hatchability rate (%) at 21 days of incubation (504 h).

Putrescine Level	% Hatchability
NC	74.18
Putrescine 0.015%	78.12
Putrescine 0.030%	71.56
Putrescine 0.060%	74.68
Putrescine 0.090%	68.75
SEM	1.901
*p*-value	0.634
P-linear	0.277
P-quadratic	0.637

NC = Negative control (0.9% NaCl saline inoculation). SEM = Standard error of the mean.

**Table 3:** Effect of putrescine in ovo on body weight (BW), percentage of yolk (%), percentage of the liver (%), breast muscle (%), and intestine (%) at hatch, and BW and intestine (%) 4 days post-hatch.

Putrescine Level									
	NC	0.015%	0.030%	0.060%	0.090%	SEM	*p*-Value	P-Linear	P-Quad.
At hatch									
BW (g) ^†^	44.51	45.24	43.62	42.71	44.23	0.402	0.353	0.352	0.234
Yolk ^‡^	14.96	12.41	13.54	11.13	11.85	0.587	0.289	0.082	0.352
Liver ^‡^	2.40	2.56	2.53	2.63	2.70	0.066	0.686	0.313	0.980
Breast ^‡^	2.99	3.00	2.89	2.80	2.81	0.060	0.879	0.699	0.934
Intestine ^‡^	4.03	4.91	4.86	4.95	4.79	0.179	0.489	0.375	0.267
4 days post-hatch									
BW (g) ^§^	97.16	91.23	96.73	97.86	97.23	1.610	0.723	0.536	0.914
Intestine ^¶^	14.48	14.88	13.19	14.73	13.64	0.228	0.054	0.338	0.948

NC = Negative control (0.9% NaCl saline inoculation). SEM = Standard error of the mean. ^†^ BW= Body weight of 6 animals per treatment euthanized for data collection at hatch. ^‡^ Weight of the tissue in relation the body weight of 6 animals per treatment euthanized for data collection at hatch. ^§^ BW = Body weight of 6 animals per treatment euthanized for data collection at 4 days post-hatch. ^¶^ Weight of the tissue in relation the body weight of 6 animals per treatment euthanized for data collection at 4 days post-hatch.

**Table 4:** Effect of putrescine in ovo on liver and breast glycogen levels (mg/g) of hatchlings.

Putrescine Level	Liver Glycogen (mg/g)	Muscle Glycogen (mg/g)
NC	6.406	1.737
Putrescine 0.015%	6.318	2.245
Putrescine 0.030%	7.311	1.981
Putrescine 0.060%	4.958	1.967
Putrescine 0.090%	3.793	1.221
SEM	0.413	0.123
*p*-value	0.034	0.084
P-linear	0.017 ^1^	0.134
P-quadratic	0.064	0.022 ^2^

NC = Negative control (0.9% NaCl saline inoculation). SEM = Standard error of the mean. ^1^ Linear equation: Liver glycogen = 8.392 − 0.6586 dose; R^2^ = 0.1753. ^2^ Quadratic equation: Muscle glycogen = 0.0956 + 1.160 dose − 0.1613 dose^2^; R^2^ = 0.2446.

**Table 5:** Effect of putrescine in ovo on villus height (VH), crypt depth (CD), and number of goblet cells (GC) of the jejunum in broilers at hatch and 4 days of age.

	At Hatch			At 4 Days Post-Hatch		
Putrescine Level	VH ^†^	CD ^‡^	GC (n^o^ in 200 µm) ^§^	VH ^†^	CD ^‡^	GC (n^o^ in 200 µm) ^§^
NC	287.20	41.65	14.21	441.25	126.54	22.60
Putrescine 0.015%	307.62	57.26	14.26	451.35	125.95	25.36
Putrescine 0.030%	307.82	61.40	16.07	484.50	115.73	24.21
Putrescine 0.060%	332.98	71.58	17.72	456.35	105.65	25.46
Putrescine 0.090%	318.42	72.20	17.91	418.84	114.44	27.66
SEM	3.130	1.110	0.014	5.10	1.684	0.030
*p*-value	<0.001	<0.001	<0.001	<0.001	<0.001	<0.001
P-linear	<0.001 ^1^	<0.001	<0.001 ^3^	0.232	<0.001 ^5^	<0.001 ^6^
P-quadratic	0.063	<0.001 ^2^	0.949	<0.001 ^4^	0.084	0.840

NC = Negative control (0.9% NaCl saline inoculation). SEM = Standard error of the mean. ^†^ VH = The villus height was measured from the base, starting immediately after the higher section of the crypt, to the top. ^‡^ CD = The crypt depth was measured from the base of crypt to the crypt:villus transition region. ^§^ GC = The goblet cells were counted along 200 µm of the intermediate section. At hatch: ^1^ Linear equation: VH = 275.7 + 8.780 dose; R^2^ = 0.0526. ^2^ Quadratic equation: CD = 3.686 + 23.17 dose − 1.987 dose^2^; R^2^ = 0.3166. ^3^ Linear equation: GC = 11.69 + 1.087 dose; R^2^ = 0.1429. At 4 days post-hatch: ^4^ Quadratic equation: VH = 309.9 + 85.46 dose − 11.18 dose^2^; R^2^= 0.0491. ^5^ Linear equation: CD = 135.5 − 4.451 dose; R^2^ = 0.0467. ^6^ Linear equation: GC = 20.97 + 1.021 dose; R^2^ = 0.0767.

**Table 6:** Effect of putrescine in ovo on villus height (VH), crypt depth (CD), and number of goblet cells (GC) of the ileum in broilers at hatch and 4 days of age.

	At Hatch			At 4 Days Post-Hatch		
Putrescine Level	VH ^†^	CD ^‡^	GC (n^o^ in 200 µm) ^§^	VH ^†^	CD ^‡^	GC (n^o^ in 200 µm) ^§^
NC	220.60	52.88	12.89	288.98	96.23	25.88
Putrescine 0.015%	262.73	59.88	15.88	303.00	96.11	29.42
Putrescine 0.030%	263.28	67.43	14.64	290.50	83.93	32.45
Putrescine 0.060%	244.08	66.15	15.79	333.68	97.45	28.56
Putrescine 0.090%	256.45	68.61	15.40	308.18	85.25	30.64
SEM	2.779	1.069	0.013	3.480	1.100	0.333
*p*-value	<0.001	<0.001	<0.001	<0.001	<0.001	<0.001
P-linear	0.006	<0.001	<0.001	≤0.001 ^4^	0.008 ^5^	<0.001
P-quadratic	<0.001 ^1^	0.034 ^2^	0.023 ^3^	0.321	0.868	<0.001 ^6^

NC = Negative control (0.9% NaCl saline inoculation). SEM = Standard error of the mean. ^†^ VH = The villus height was measured from the base, starting immediately after the higher section of the crypt, to the top. ^‡^ CD = The crypt depth was measured from the base of crypt to the crypt: villus transition region. ^§^ GC = The goblet cells were counted along 200 µm of the intermediate section. At hatch: ^1^ Quadratic equation: VH = 148.9 + 50.61 dose − 5.662 dose^2^; R^2^ = 0.0633. ^2^ Quadratic equation: CD = 30.00 + 14.00 dose − 1.278 dose^2^; R^2^ = 0.0968. ^3^ Quadratic equation: GC = 8.563 + 2.997 dose- 0.3130 dose^2^; R^2^ = 0.0596. At 4 days post-hatch: ^4^ Linear equation: VH = 277.2 + 6.909 dose; R^2^= 0.0808. ^5^ Linear equation: CD = 100.0 − 2.063 dose; R^2^ = 0.1092. ^6^ Quadratic equation: GC = 16.11 + 6.477 dose − 0.7017 dose^2^; R^2^ = 0.0864.

**Table 7:** Effect of putrescine in ovo on feed intake (FI), body weight gain (BWG), and feed conversion ratio (FCR) of broilers at 7 d, 14 d and 21 d of age.

Putrescine Level									
	NC	0.015%	0.030%	0.060%	0.090%	SEM	*p*-Value	P-Linear	P-Quad.
1–7 days									
FI (g)	168.76	160.85	169.90	167.26	163.05	2.614	0.742	0.740	0.747
BWG (g)	133.39	122.09	132.66	133.03	128.85	2.320	0.454	0.920	0.888
FCR (g/g)	1.269	1.271	1.290	1.240	1.294	0.0124	0.698	0.827	0.565
1–14 days									
FI (g)	530.53	514.39	550.60	543.50	541.28	7.038	0.528	0.373	0.576
BWG (g)	408.30	393.22	414.68	414.54	414.43	6.873	0.849	0.508	0.915
FCR (g/g)	1.301	1.309	1.338	1.317	1.311	0.012	0.935	0.889	0.488
1–21 days									
FI (g)	1173.33	1114.04	1187.20	1186.25	1181.61	12.383	0.243	0.326	0.899
BWG (g)	855.43	816.66	882.20	875.79	864.24	10.086	0.242	0.343	0.522
FCR (g/g)	1.346	1.365	1.349	1.355	1.369	0.006	0.833	0.449	0.793

NC = Negative control (0.9% NaCl saline inoculation). SEM = Standard error of the mean.

## Data Availability

The data presented in this study are available on request from the corresponding author.
